# MMP-8 in Periodontal Sites of Postpartum and without-Any-Pregnancy Women

**DOI:** 10.3390/ijerph21060739

**Published:** 2024-06-06

**Authors:** Karyne Martins Lima, Keiko Aramaki Abreu Calado, Adriana de Fátima Vasconcelos Pereira, Mayara Cristina Pinto da Silva, Fernanda Ferreira Lopes

**Affiliations:** 1 Postgraduate Program in Dentistry, Federal University of Maranhão, São Luís CEP 65080-805, Brazil; fernanda.ferreira@ufma.br; 2 Postgraduate Program in Adult Health, Federal University of Maranhão, São Luís CEP 65080-805, Brazil; keiko_aramaki@hotmail.com (K.A.A.C.); mayara.silva@ufma.br (M.C.P.d.S.); 3 Teacher of the Department of Dentistry II, Federal University of Maranhão, São Luís CEP 65080-805, Brazil; adriana.vasconcelos@ufma.br

**Keywords:** matrix metalloproteinase 8, postpartum women, periodontal diseases

## Abstract

The hypothesis that physiological changes in women can affect periodontal tissues is the subject of this study, and inflammatory markers such as matrix metalloproteinase-8 can measure susceptibility to inflammation. The study aimed to analyze MMP-8 levels in periodontal sites of postpartum women and women without a history of pregnancy, comparing health parameters and periodontal disease. This is a case–control study with 40 participants, 20 cases (women in the postpartum period) and 20 controls (women without any pregnancy), who underwent clinical periodontal examination and the collection of crevicular gingival fluid. The ELISA test was used to detect MMP-8 levels. Postpartum women had worse periodontal parameters, such as bleeding index on probing, number of sites with CAL ≥ 3, and fewer teeth present. In the group of women without a history of pregnancy, a significantly lower MMP-8 level was observed in healthy sites and a higher one was observed in periodontal pockets (*p* < 0.01). In contrast, in postpartum women, MMP-8 levels were elevated in both healthy sites and periodontal pockets (*p* > 0.01). The MMP-8 levels in gingival fluid appear to be related to periodontal clinical parameters and may be a possible marker of enzymatic changes involved in periodontal tissue destruction in postpartum women.

## 1. Introduction

There is evidence that periodontal diseases can induce systemic inflammatory responses due to an increase in leukocytes and the release of cytokines, enzymes, and plasma fibrinogen [1,2]. Therefore, it can cause or worsen cardiovascular diseases and induce premature birth, low birth weight, and kidney disease [3,4].

Women are more susceptible to hormonal changes, especially during puberty, pregnancy, and menopause [5]. These hormonal changes can induce an immunological response in the oral microbiota and cause vascular and gingival changes that may contribute to increased gingival inflammation [6]. During pregnancy, there is an increased risk for women to develop periodontal diseases due to high levels of estrogen and progesterone, which are related to an imbalance in the oral microbiota and can trigger an exacerbated inflammatory condition [7].

Neutrophils are directly influenced by these hormones, with an increase in quantity in the gingival sulcus or periodontal pocket, and are considered a source of a variety of pro-inflammatory cytokines, molecules, and proteases, including matrix metalloproteinases (MMPs) [8].

MMP levels are altered during pregnancy due to hormonal activity and are related to adverse events during pregnancy such as premature cervical dilation, membrane rupture, presence of microbial in the amniotic cavity, and premature birth [9,10]. Its excessive or unbalanced activity has been associated with the progression of periodontal diseases, myocardial infarction, aneurysm, and tumor metastasis, among other diseases [11,12].

MMP-8 (metalloprotrainase-8) stimulates the degradation of type I collagen, the interstitial type that is dominant in periodontal tissues, which is considered to be one of the most promising biomarkers for the early diagnosis of periodontitis [13,14,15]. Elevated levels of MMP-8 in gingival crevicular fluid and salivary fluid associated with periodontal disease have already been reported [16,17,18,19].

Some studies found an increase in MMP-8 levels during the gestational and postpartum period [20,21]. Gursoy and collaborators [22] indicated higher MMP-8 levels in pregnancy and lower ones in the postpartum period, while Erhles and collaborators [23] found no significant difference between pregnant women and those without any pregnancy in terms of their MMP-8 levels. Research that addresses the periodontal profile of patients with periodontitis used crevicular gingival fluid by the evaluation of biomarkers evaluated in healthy sites and diseased sites/pockets [24,25].

A greater understanding of the relationship between MMP-8 levels in gingival crevicular fluid in postpartum and non-pregnant women with periodontitis is not available in the current studies. Therefore, the objective of this study is to analyze MMP-8 levels in periodontal sites of postpartum women and women without pregnancy, comparing health parameters, possible hormonal changes due to pregnancy, and periodontal disease, to verify whether MMP-8 can be a possible marker of enzymatic changes involved in the destruction of periodontal tissue in postpartum women.

## 2. Materials and Methods

### 2.1. Study Design

This is a case–control study, using a convenience sample, whose participants agreed to answer a questionnaire and undergo a periodontal examination. The clinical examination and data collection of women with immediate postpartum were carried out at the bedside on the 3rd floor (Obstetrics Wing) of the Maternal and Child Unit, and the examination of collection for women without any pregnancy were carried out in the Dental Outpatient Clinic (1st floor) in the Presidente Dutra Unit, both within the Federal University of Maranhão.

### 2.2. Sample Selection

Inclusion criteria: Adult women (18–37 years of age) with at least 20 teeth, excluding third molars, were eligible for research. A total of 40 participants were included into 2 groups: 20 women in the immediate postpartum period (1st to 10th day postpartum) and 20 women without any pregnancy.

All patients were diagnosed with periodontitis, following the criteria of clinical attachment level (CAL) ≥ 3 mm with probing pocket depth (PPD) > 3 mm detectable in ≥2 non-adjacent teeth and the presence of bleeding on probing [26].

Exclusion criteria: Women with the presence of a systemic condition that could influence periodontal condition (diabetes mellitus, heart disease, neurological diseases, obesity, viral and bacterial infections); smokers and alcoholics; those who used fixed orthodontic appliances; mouth breathers; women over the age of 37 due to the increased risk of premature birth; chronic use of antibiotics or non-steroidal or steroidal anti-inflammatory drugs in the 6 months before the research; use of antihypertensive, anticonvulsant, or immunosuppressant medications or any other medications that are known to have the possibility of resulting in gingival enlargement.

Furthermore, for the group of postpartum women, those who were more than 10 days postpartum and had twin pregnancies were removed. For the group of women without any pregnancy, those who had previously had abortions were excluded.

### 2.3. Data Collection

Clinical phase: Data were collected on oral hygiene habits (use of tooth brushing after meals and its frequency, use of dental floss and its frequency, and visits to the dentist in the last year) and on habits regarding history of tobacco and alcohol use (ever tried or not). Information about the gestational period of the postpartum women was confirmed in the medical records analyzed before the first approach.

For the periodontal examination, a clinical kit was used, containing mouth mirror number 5, clinical tweezers, Williams’ periodontal probe, and a flashlight attached to the examiner’s head.

After screening for exclusion criteria and initial clinical examination, the participants underwent a periodontal examination to confirm or not the diagnosis of periodontitis. All participants had a healthy periodontal site [24,25] and were diagnosed with periodontitis because they showed CAL ≥ 3 mm with probing pocket depth > 3 mm detectable in ≥2 non-adjacent teeth and the presence of bleeding on probing [26]. In this way, samples from two different sites/teeth (gingival sulcus and periodontal pocket) were obtained and stored individually for later analysis.

### 2.4. Periodontal Assessment

The participants underwent periodontal evaluation by 2 previously calibrated evaluators (Kappa Index = 0.7) using the following clinical parameters:

- Probing pocket depth (PPD): measured with a millimeter periodontal probe, determined as the distance from the gingival margin to the bottom of the sulcus or periodontal pocket.
- Bleeding on probing (BOP): the occurrence of probing bleeding was recorded 10 s after removing the millimeter probe inside the groove or periodontal pocket [19].
- Clinical attachment level (CAL): distance in millimeters between the cementoenamel junction and the bottom of the groove or periodontal pocket [27].

A full-mouth examination was performed, and measurements were obtained in six sites (mesio-buccal; mid-buccal; disto-vestibular; mesio-lingual; mid-lingual; and disto-lingual) of each tooth present in the oral cavity, except for the third molars.

### 2.5. Gingival Fluid Collection

During material collection, the site involved was properly isolated and dried with sterilized cotton rolls. The supragingival portion of the bacterial biofilm was removed by Gracey currete to then obtain samples of gingival fluid by placing 2 cones of absorbent paper (Dentsply^®^ brand nº 25) per healthy and diseased site to obtain an adequate volume of periodontal fluid. The paper cones were then placed in dry and sterile microcentrifuge tubes (eppendorf), which were coded for identification. The samples were stored for later analysis at −800 °C.

### 2.6. Sample Preparation

The samples were prepared by adding 150 μL of PBS, sonicating for 10 s, vortexing for 1 min, and then centrifuging for 10 min at 13,200 rpm cold. The samples were analyzed immediately for the detection of MMP-8 levels using an ELISA test (enzyme-linked immunosorbent test) following the manufacturer’s R&D SYSTEMS protocol. The concentrations of MMP-8 present in gingival crevicular fluid were expressed in ng/mL.

### 2.7. ELISA Test

#### 2.7.1. Plate Preparation

1. The capture antibody was diluted to the working concentration in PBS without carrier protein. Each well was coated with 100 μL of the capture antibody. The plate was sealed and incubated overnight at room temperature.
2. The next day, the wells were aspirated and washed with buffer solution, repeating the process twice for a total of three washes. Each well was washed with wash solution (400 μL) using a squirt bottle, manifold dispenser, or automatic washer. Complete removal of liquid at each step was essential for good performance. After the last wash, it was necessary to remove any remaining wash buffer by vacuuming or inverting the plate and rubbing it against paper towels.
3. Plates were blocked by adding 300 μL of reagent diluent to each well. These were incubated at room temperature for at least 1 h.
4. Aspiration/washing was repeated as in step 2. Then, the plates were ready for sample addition.

#### 2.7.2. Assay Procedure

1. A total of 100 μL of sample or standard was added to the reagent diluent or an appropriate diluent per well. It was covered with adhesive tape and incubated for 2 h at room temperature.
2. Aspiration/washing was repeated as in step 2 of the plates’ preparation.
3. A total of 100 μL of detection antibody, diluted in NGS diluent reagent, was added to each well. This was covered with new adhesive tape and incubated for 2 h at room temperature.
4. Aspiration/washing was repeated as in step 2 of plate preparation.
5. In total, 100 μL of the streptavidin–HRP working dilution was added to each well. The plate was covered and incubated for 20 min at room temperature. Placing the plate in direct light was avoided.
6. Vacuuming/washing was repeated as in step 2.
7. A total of 100 μL of substrate solution was added to each well. These were incubated for 20 min at room temperature, and we avoided placing the plate in direct light.
8. In total, 50 μL of stop solution was added to each well. We gently tapped the plate to ensure thorough mixing.
9. The optical density of each well was determined immediately using a microplate reader set at 450 nm.

### 2.8. Statistical Analysis

Data were tabulated in the Excel^®^ program (Microsoft Excel^®^, Washington, DC, USA) and analyzed using the PRISMA Program (version 5.1) In numerical variables, the Shapiro–Wilk test was used to assess the normality of the distribution and, subsequently, the Mann–Whitney U statistical test or the unpaired T test was used to verify the association between the study groups. The significance level for all the statistical tests was 5%.

## 3. Results

Data from 40 women, 20 in the immediate postpartum period and 20 without any pregnancy, revealed that 90% of participants reported brushing their teeth after meals, with no significant association between the groups with *p* = 0.1060. In the group of postpartum women, 55% reported brushing their teeth two or fewer times a day, 60% did not use dental floss, and 80% did not visit the dentist in the last year. However, in the group of women without any pregnancy, 70% brushed three or more times a day, 85% flossed, and 60% visited the dentist in the last year. There was a statistically significant difference between the groups about the visit to the dentist in the last year (*p* = 0.0128), the use of dental floss (*p* = 0.0079), and their frequency of daily brushing (*p* < 0.0001), as can be seen in Table 1.

Regarding previous experience with the use of alcohol, there was a statistically significant difference (*p* = 0.0008) where most of the group without any pregnancy (90%) reported having already consumed alcoholic beverages, while 65% of the postpartum women had not. The majority of both groups (90%) reported never having used tobacco (Table 1).

Table 2 presents the average periodontal parameters of the 40 participants. Postpartum women had worse periodontal parameters, such as a smaller number of teeth present, greater number of bleeding sites, and higher Probing Bleeding Index (ISS) compared to the group of participants without any pregnancy.

**Figure 1:**
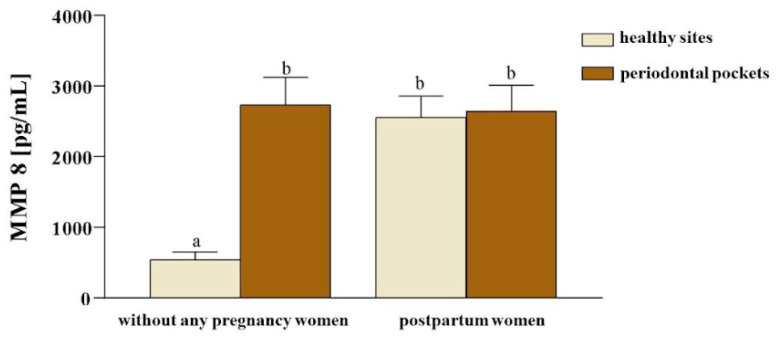
Distribution of MMP-8 levels in the no pregnancy and postpartum women groups. Different letters indicate that there was a statistical difference between the means at 5% significance. a & b: *p* < 0.05.

The level of MMP-8 in the sample, consisting of gingival fluids from healthy sites and periodontal pockets of women without any pregnancy and women who have recently given birth, is expressed in Table 3 and illustrated in Figure 1.

A significantly lower level of MMP-8 was observed in healthy sites of women without any pregnancy and higher in periodontal pockets (*p* < 0.01), while in postpartum women, the MMP-8 levels were elevated both in the sites and periodontal pockets (*p* > 0.01).

## 4. Discussion

Changes in clinical parameters present in periodontal disease and MMP-8 levels in women who have recently given birth and without any pregnancy were the object of study in this research. It showed that the MMP-8 levels in gingival fluid appear to be related to periodontal clinical parameters and may be a possible marker of enzymatic changes involved in periodontal tissue destruction in postpartum women.

Periodontitis is defined as a multifactorial chronic inflammatory disease associated with dysbiotic biofilm and characterized by the progressive destruction of the dental attachment structure, initiated by a biofilm with a predominance of Gram-negative anaerobic microorganisms and mediated by the host’s inflammatory response [28]. And during pregnancy, gingivitis is very common [29]. Although it is established that periodontal pathogens in oral biofilm are the main triggering agents for periodontal disease, it has become clear from the evaluation of recent studies that host inflammation, including biomarkers such as MMP-8, plays an important role as a pathological mechanism for periodontal diseases and systemic diseases [11,12,30].

In a longitudinal study [Santana and colleagues], the pre-pregnancy Body Mass Index (BMI) was significantly associated with increased C-reactive proteins levels from pregnancy to the beginning of the postpartum period among Brazilian pregnant women with periodontitis; therefore, it is necessary to investigate whether predictors of changes in inflammatory markers can be used as prognostic factors for gestational outcomes, thus collaborating with the findings of this research in which the postpartum state would increase inflammatory biomarkers [31].

Regarding the periodontal condition, it was detected that the rate of bleeding on probing was higher in postpartum women (*p* = 0.0339), showing that women who had already become pregnant showed frequent clinical signs of gingival inflammation. A possible explanation for this result may be the participants’ low frequency of tooth brushing, in which only 35% of postpartum women reported brushing their teeth three or more times a day, highlighting the poor oral hygiene practiced by women during pregnancy, as already previously detected [29].

The postpartum women had worse oral parameters, revealing a less favorable oral condition and that they could be in the early stages of periodontal disease. This may be related to poor oral hygiene in the interproximal region of women who have already become pregnant, since 60% of women who have recently given birth reported not using dental floss, confirming a common practice among pregnant women [32,33]. When observing these results, oral health services need to be routinely integrated into prenatal services for all pregnant women [34] to detect the onset of periodontal disease early and control and plan the appropriate treatment. Furthermore, in this research, in postpartum women, MMP-8 levels were elevated both in periodontal sites and periodontal pockets (*p* > 0.01), so it may be a possible marker of enzymatic changes involved in periodontal tissue destruction in pregnancy.

Tooth loss results in more advanced stages of periodontal disease [26], being a clinical parameter of the study. Women who had never been pregnant had a significantly greater number of teeth than women who had recently given birth (*p* = 0.0024); however, in this study, it was not possible to identify the cause of tooth loss. However, this result confirms that pregnant women may be more negligent with their oral health due to the lack of professional guidance, difficult access to dental care, popular beliefs, and the fear that some dental surgeons still have to treat pregnant women [6,35].

In this study, most postpartum women (80%) reported not having visited the dentist in the last year. This finding is confirmed in several studies [6,33,34,35,36], which showed that it is common to abstain from dental care during pregnancy. Furthermore, a study demonstrated that multiparous women with young children may have less time, less energy, and fewer financial resources to invest in dental care, interfering with attendance at dentist appointments [6].

The behavioral habits covered in this study were history regarding the use of alcohol and tobacco, substances that can generate chemical dependence in their users, in addition to being risk factors for the development of halitosis [37]. When asked about their experience regarding tobacco use, 90% of all participants reported not using tobacco, confirming the significant drop in the percentage of smokers in Brazil over the last three decades. This is probably due to awareness campaigns in the media, municipal laws that restrict smoking areas, and publicity about the consequences caused by smoking. However, 90% of women who have never been pregnant reported experience with alcohol, showing a growing trend in alcohol consumption [38].

A significantly lower level of MMP-8 was observed only in the periodontal sites of women without any pregnancy (*p* < 0.01); however, Erlhes et al. [23] found no significant differences in MMP-8 levels in the gingival fluid of non-pregnant and pregnant women, although the levels were higher in the latter group. A possible explanation for the divergence in results may lie in the research methodology, as our study not only made a comparison between groups but also between the periodontal site and pocket of the same individual participating in the research.

Matrix metalloproteinases (MMPs) are a family of zinc-dependent endopeptidases with an important role in pathological and physiological remodeling [11,12], making the findings of the present study relevant since the MMP-8 levels were elevated both in healthy sites as well as in periodontal pockets in women in the immediate postpartum period (*p* > 0.01).

Some studies have suggested that elevated MMP-8 levels in saliva, amniotic fluid, and serum during preterm and full-term labor may be associated with the spontaneous rupture of membranes and preeclampsia in pregnancy, thus supporting its role in the development and progression of adverse events in pregnancy [39,40]. However, a systematic review concluded that there was no way to determine the effectiveness of non-surgical periodontal treatment through MMP-8 levels in gingival crevicular fluid, but it was possible to observe a decrease in probing depth in the population after 3 months [41]. In our study, periodontal treatment was not performed; however, our results showed that MMP-8 may be related to changes in periodontal parameters for diagnostic use. However, both MMP-8 and MMP-9 are valuable diagnostic tools in treating periodontitis, and the determination of crevicular MMP-8 might represent a rapid and accurate diagnostic method, so future studies should focus on implementing more accessible methods of chairside testing in order to reduce the prevalence of this disease [42].

The present findings suggest that high levels of MMP-8 in crevicular fluid reflect the inflammatory load during pregnancy, and these high levels may result in changes in gingival tissues. Therefore, MMP-8 levels may be useful for screening, early diagnosis, and monitoring changes in gingival, oral, and systemic tissues during pregnancy through the MMP diagnostic tools available during dental care [43,44]. And biomarkers such as MMP-8 can assist in both the staging and classification of periodontitis, so in the future, studies should focus on implementation and more efficient in-person testing that could be used in daily clinical practice [45].

This research presents as strengths the clinical data analyzed based on the new scheme for the Classification of Periodontal and Peri-implant Diseases and Conditions [28], including the frequency of bleeding sites on probing [26], through a full-mouth periodontal examination, which allows you to define the periodontal treatment plan. However, the limitations of the research include the impossibility of classifying the severity, progression, and complexity of the disease, due to the lack of radiographic information on bone loss and the identification of tooth loss due to periodontitis. More studies are needed to corroborate the preliminary data of the present study.

Over time, robust evidence has shown a relationship between periodontal disease and systemic diseases. Although its mechanism has not been elucidated, the possibility of causing adverse events during pregnancy keeps researchers and clinical professionals alert about the possibility of affecting maternal and child health. Therefore, dental prenatal care becomes an excellent strategy for achieving comprehensive, quality, and easily accessible care.

## 5. Conclusions

MMP-8 levels in gingival fluid appear to be related to periodontal clinical parameters and may be a possible marker of enzymatic changes involved in periodontal tissue destruction in postpartum women.

## Figures and Tables

**Table 1:** Behavioral habits of postpartum women and those without any pregnancy (*n* = 40).

	Group			
	Postpartum Women	No Pregnancy	*p* *	Total
**Behavioral habits**	20 (100%)	20 (100%)		40 (100%)
**Brushing after meals**				
Yes	16 (80.00%)	20 (100.00%)	*p* = 0.1060	36 (90.00%)
No	4 (20.00%)	0		4 (10.00%)
**Daily brushing frequency**				
Two or fewer times	12 (55.00%)	6 (30.00%)	*p* = 0.056 *	18 (45.00%)
Three or more times	8 (35.00%)	14 (70.00%)		22 (55.00%)
**Flossing**				
Yes	8 (40.00%)	17 (85.00%)	*p* = 0.0079 *	25 (62.50%)
No	12 (60.00%)	3 (15.00%)		15 (37.50%)
**Visit to the dentist**				
Yes	4 (20.00%)	12 (60.00%)	*p* = 0.0128 *	14 (40.00%)
No	16 (80.00%)	8 (40.00%)		24 (60.00%)
**History of alcohol**				
Yes	7 (35.00%)	18 (90.00%)	*p* = 0.0008	25 (62.50%)
No	13 (65.00%)	2 (10.00%)		15 (37.50%)
**Past smoking history**				
Yes	2 (10.00%)	2 (10.00%)	*p* = 1.0000	4 (10.00%)
No	18 (90.00%)	18 (90.00%)		36 (90.00%)

* Fisher’s exact test or Chi-square test (α = 0.05).

**Table 2:** Mean and standard deviation of oral parameters in women who have recently given birth and those without any pregnancy.

Oral Parameters	Group		*p*-Value *
	Postpartum Women (n = 20)	No Pregnancy (n = 20)	
Number of bleeding sites	29.10 ± 18.74	19.85 ± 10.71	0.0632
Bleeding on probing (POB)	26.80 ± 16.78	17.10 ± 9.67	0.0339 *
Number of teeth	26.50 ± 1.64	27.70 ± 0.93	0.0024 *

* Mann–Whitney test (α = 0.05).

**Table 3:** Summary measurements of the level of metalloproteinase 8 (MMP-8) in pg/mL at the healthy site and in the periodontal pocket of women who have recently given birth and those who are not pregnant.

Summary Measurements of MMP-8 Level in pg/mL	Groups			
	Postpartum Women		No Pregnancy	
	Place	Handbag	Place	Handbag
Minimum	104	145.3	30.25	120.3
Maximum	4280	4898	1742	4895
Variance	4176	4753	1711	4775
Average	2560	2653	540.3	2735
Standard deviation	1315	1652	449.6	1596

## Data Availability

The authors confirm that data supporting this study’s findings are available within the article.
